# Nutraceutical Capsules LL1 and Silymarin Supplementation Act on Mood and Sleep Quality Perception by Microbiota–Gut–Brain Axis: A Pilot Clinical Study

**DOI:** 10.3390/nu16183049

**Published:** 2024-09-10

**Authors:** Aline Boveto Santamarina, Victor Nehmi Filho, Jéssica Alves de Freitas, Lucas Augusto Moysés Franco, Joyce Vanessa Fonseca, Roberta Cristina Martins, José Antônio Orellana Turri, Bruna Fernanda Rio Branco da Silva, Arianne Fagotti Gusmão, Eloísa Helena Ribeiro Olivieri, José Pinhata Otoch, Ana Flávia Marçal Pessoa

**Affiliations:** 1 Laboratório de Produtos e Derivados Naturais, Laboratório de Investigação Médica-26 (LIM-26), Departamento de Cirurgia, Faculdade de Medicina de São Paulo, Universidade de São Paulo, São Paulo 01246-903, SP, Brazil; alinesantamarina@gmail.com (A.B.S.); victor@nehmi.com.br (V.N.F.); pinhata@usp.br (J.P.O.); 2 Pesquisa e Desenvolvimento Efeom Nutrição S/A, São Paulo 03317-000, SP, Brazil; 3 Laboratório de Parasitologia Médica (LIM-46), Departamento de Doenças Infecciosas e Parasitárias, Instituto de Medicina Tropical de São Paulo, Universidade de São Paulo, São Paulo 05403-000, SP, Brazil; 4 Laboratório de Investigação Médica em Protozoologia, Bacteriologia e Resistência Antimicrobiana (LIM-49), Departamento de Doenças Infecciosas e Parasitárias, Instituto de Medicina Tropical de São Paulo, Universidade de São Paulo, São Paulo 05403-000, SP, Brazil; 5 Grupo de Pesquisa em Economia da Saúde, Departamento de Ginecologia e Obstetrícia, Faculdade de Medicina, Universidade de São Paulo, São Paulo 01246-903, SP, Brazil; 6 Laboratório Interdisciplinar em Fisiologia e Exercício, Universidade Federal de São Paulo (UNIFESP), Santos 11015-020, SP, Brazil; 7 International Research Center, A.C. Camargo Cancer Center, São Paulo 01508-010, SP, Brazil; 8 Hospital Universitário da Universidade de São Paulo, Faculdade de Medicina de São Paulo, Universidade de São Paulo, São Paulo 05508-000, SP, Brazil

**Keywords:** gut-brain axis, prebiotics, dysbiosis, inflammation, microbiota, sleep

## Abstract

Stress, unhealthy lifestyle, and sleep disturbance worsen cognitive function in mood disorders, prompting a rise in the development of integrative health approaches. The recent investigations in the gut–brain axis field highlight the strong interplay among microbiota, inflammation, and mental health. Thus, this study aimed to investigate a new nutraceutical formulation comprising prebiotics, minerals, and silymarin’s impact on microbiota, inflammation, mood, and sleep quality. The study evaluated the LL1 + silymarin capsule supplementation over 180 days in overweight adults. We analyzed the fecal gut microbiota using partial 16S rRNA sequences, measured cytokine expression via CBA, collected anthropometric data, quality of life, and sleep questionnaire responses, and obtained plasma samples for metabolic and hormonal analysis at baseline (T0) and 180 days (T180) post-supplementation. Our findings revealed significant reshaping in gut microbiota composition at the phylum, genus, and species levels, especially in the butyrate-producer bacteria post-supplementation. These changes in gut microbiota were linked to enhancements in sleep quality, mood perception, cytokine expression, and anthropometric measures which microbiota-derived short-chain fatty acids might enhance. The supplementation tested in this study seems to be able to improve microbiota composition, reflecting anthropometrics and inflammation, as well as sleep quality and mood improvement.

## 1. Introduction

Stress, sedentarism, overweight, and unhealthy diet are triggering factors for cognitive impairment in generalized anxiety disorder or major depressive disorder [1]. Sleep disturbance, a core symptom of mood disorders, may exacerbate cognitive impairment linked to reduced psychosocial function and lower quality of life [2].

Neuropsychiatric disorders, particularly mood and sleep disorders, are intricately linked with inflammation. Growing evidence suggests a reciprocal relationship between anxiety, depression, and inflammation, amplifying each other [3]. Elevated inflammation correlates with the advancement or severity of mood disorders. Specifically, heightened C-reactive protein and interleukin-6 (IL-6) levels have been linked to the onset of depressive and anxiety symptoms, whereas remission is associated with the normalization of inflammatory markers [4].

While the signaling pathways remain debated, abnormal gut microbiota and serum proinflammatory factors are suggested as contributors to central immune activation [5]. The gut microbiota influences brain health and diseases, highlighting the connection between the gut microbiota and neuroinflammation through the gut–brain axis [5]. A balanced microbiota comprises psychobiotics, such as Lactobacillus and Bifidobacterium, which exert positive effects on mental health. These bacteria significantly influence metabolism and central nervous system function through the gut–brain axis, through neuronal, endocrine, and immune mechanisms [6,7].

To enhance long-term quality of life and mood disorders, non-pharmacological approaches targeting sleep and diet quality have been explored as a promising area in promoting the gut–brain axis balance [8]. Silymarin (*Silybum marianum* (L.) Gaertn.) for instance, can elevate dopamine levels, influencing mood and sleep quality positively [9]. Chromium and selenium are essential micronutrients for the immune response by immunomodulation in lymphocytes, macrophages, and cytokines [10,11]. Additionally, minerals like zinc and magnesium serve as cofactors in serotonin synthesis, a hormone crucial for well-being and sleep regulation [12,13]. Serotonin, produced not only by the pineal gland but also by the large intestine, underscores the importance of maintaining a balanced intestinal microbiota [14]. Given modern dietary habits often lacking in fiber and essential minerals [14], continuous intake of prebiotics such as β-glucans [15], GOS (galactooligosaccharides) [16], and FOS (fructooligosaccharides) [17] can enhance mineral absorption and intestinal health, and fortify the gut–brain axis [18].

Thus, the present study investigates the effects of 180-day supplementation with LL1 (Long Life 1) nutraceutical compositions containing prebiotics (β-glucans, GOS, and FOS), minerals (zinc, magnesium chromium, and selenium), associated with the herbal medicine (Silymarin extract—*Silybum marianum* (L.) Gaertn), [19] as modulators of the brain–gut axis, providing better mood and sleep quality.

## 2. Materials and Methods

### 2.1. Ethics, Recruitment, and Experimental Design

The Ethics Committee for the Analysis of Research Projects (CAPPesq) and HC-FMUSP Research Ethics Committee approved this protocol under the number 5.365.566. This research followed the Helsinki World Medical Declaration [20] and the relevant guidelines and regulations. This study is registered in the Clinical Trial ID 39984320.5.0000.0068 (ClinicalTrials.gov—23 March 2021) and in the Brazilian National System of Genetic Registration—SisGen (number AC29D69). All volunteers read and signed a free and informed consent term before starting the study and can withdraw consent at any time.

The “Novel Nutraceutical Supplement trial” is a pilot study addressing volunteers recruited by online invitation from March 2021 to June 2021. This study included healthy adult people of any sex, with a BMI between 18.5 and 34.9 kg/m^2^, and no recent changes in lifestyle. The exclusion criteria were as follows: insulin injection use, corticoids, and non-steroidal anti-inflammatory drugs for 15 days; AIDS or hepatitis diagnosis; pregnancy; chemotherapy treatment; and allergy to any components in the formulation. The samples and data were obtained at two points: before supplementation at day 0 (T0) and 180 days post-supplementation (T180). Volunteers were instructed to take one capsule of silymarin (*Silybum marianum* (L.) Gaertn.) extract daily, along with two LL1 capsules in the morning and two LL1 capsules in the evening every day. Neither nutritional nor physical activity interventions were performed or stimulated.

In gut microbiota research, there is an absence of an ideal placebo that does not affect gut bacteria, since traditional placebos like starch, resistant starch, cellulose, sugar, and maltodextrin can interact with the gut microbiota [21,22,23,24,25,26,27,28,29]. Due to this bias, the study was performed with a self-control design. This approach involves comparing participants’ data before and after supplementation in paired analyses.

**Figure 1:**
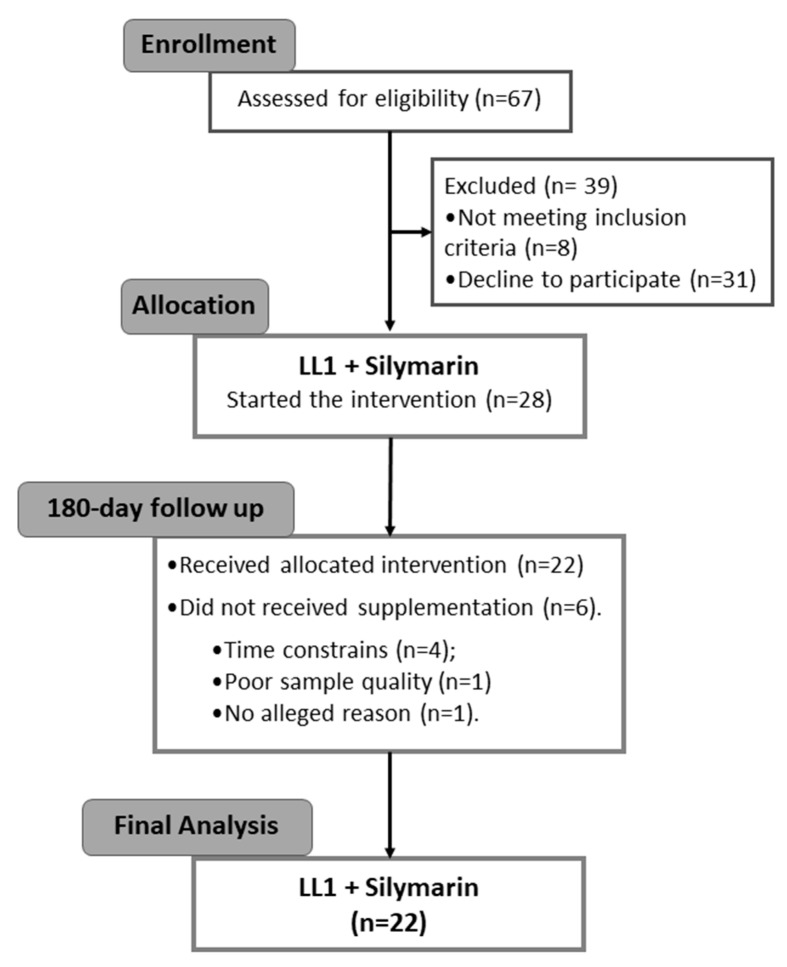
Consolidated Standards of Reporting Trials (CONSORT) flowchart of the experimental design.

We attended the Consolidated Standards of Reporting Trials (CONSORT) [30] used to build up the trial flowchart (Figure 1). The enrollment assessed 67 volunteers, of whom 39 were excluded at the initial screening (8 volunteers did not meet the inclusion criteria and 31 declined to participate); thus, 28 participants gathered baseline data. During the supplementation, 1 participant dropped out for no alleged reason, 4 left due to time constraints, and 1 volunteer was excluded due to poor sample quality. Thus, 22 participants completed the protocol.

The supplements’ composition is presented in Table 1. Their property is registered under patent number (BR 10 2020 016156 3), which can be accessed upon request. The formulation follows the European Food Safety Authority (EFSA) standards [31]. The formulations were produced by “Bioghen suplementos nutricionais LTDA” (Itap. da Serra, Brazil).

### 2.2. Dietary Intake, Physical Activity, Sleep, Mood, and Quality of Life

Participants’ dietary intake data were obtained at T0 and T180 from a three-day food diary and analyzed using DietPro software (version 6.1). Physical activity was assessed through the International Physical Activity Questionnaire (IPAQ) [32]. The Brazilian Portuguese Version of the Mini-Sleep Questionnaire (MSQ-BR) [33], the Epworth sleepiness scale (ESS) [34], and the Pittsburgh Sleep Quality Index (PSQI) [35] were applied to assess sleep quality. The participants’ quality of life was evaluated by the “World Health Organization Quality of Life instrument-short form” (WHOQoL-BREF) [36]. The Brunel Mood Scale (BRUMS) was used to screen the volunteers’ mood perception [37].

### 2.3. Anthropometrics and Biochemistry Parameter

Anthropometric measurements and plasma samples were assessed before (T0) and after (T180) supplementation. The body mass and height were measured using the Body Composition Scale 2 (Xiaomi Mi, Beijing, China) and circumferences were gauged using a plastic tape measure. Body Mass Index [BMI = body mass (kg)/height (m)^2^], waist-to-hip ratio (WHR), and waist-to-height ratio (WHtR) were calculated. Fasting plasma samples obtained between 7:00 a.m. and 9:00 a.m. underwent analysis conducted by the “Fleury Medicina e Saúde” laboratory for endocrine and metabolic parameters. The total cholesterol, high-density lipoprotein cholesterol (HDL-cholesterol), immunoglobulin M (IgM), albumin, creatinine, thyroid stimulating hormone (TSH), and thyroxine were evaluated.

### 2.4. Cytokine and Chemokine Levels

The cytokine and chemokine concentrations in plasma were analyzed by Cytometric Bead Array (CBA) in samples kept at −80 °C. The preparation of beads, standards, reagents, and samples, and the protocols for flow cytometer setup and data acquisition were performed according to the manufacturer’s instructions. The FACS Canto II flow cytometer (Becton Dickinson Holdings Pte Ltd., Franklin Lakes, NJ, USA) and commercial kits from BD^TM^ CBA for “552990—Human Chemokine (RRID AB_2868970)” and “551811—Human Inflammatory Cytokines CBA kit (RRID AB_2868941)” were used (BD Biosciences, Franklin Lakes, NJ, USA). Results were calculated in CBA Analysis Software V1.1.15 (SoftFlow, Pecs, Hungary) and were expressed in pg/mL.

### 2.5. Microbiome Analysis

Sample collection, genomic extraction, library preparation, sequencing procedures, and bioinformatic analysis were performed as previously described by Nehmi-Filho, de Freitas, et al. (2024) [38]. Briefly, the 16S rRNA gene data underwent preprocessing and diversity estimation using Quantitative Insights Into Microbial Ecology (QIIME 2) version 2020.11 [39]. The average number of sequences per sample was 62,438. The denoising step generated amplicon sequence variants (ASVs) [40], identifying 2656 ASVs. Alpha and beta diversity indices were calculated using Q2-diversity. The samples were rarefied to 20,622 sequences per sample [41] before estimating these metrics.

The taxonomic classification of ASVs was performed using the Q2-feature-classifier [42] specifically employing the naive Bayes classifier against the Greengenes 13_8 99% OTUs (Operational Taxonomic Unit) reference sequences. The microbiota composition was summarized at taxonomic levels, including species, genera, families, orders, classes, and phyla ranks. A heatmap visualization technique focusing on phyla, genera, and species was employed. The Linear Discriminant Analysis Effect Size (LEfSe) algorithm (version 1.1.2) was applied to identifying features with biological relevance. Differential abundance analysis was conducted utilizing the R (4.3.1) package DESeq2 (1.42.0).

### 2.6. Statistical Analysis

The sample size enrolled was based on a calculation determined using the G*Power software version 3.1 [43] assuming a *T*-test (Means: Difference between two dependent means–matched pairs) with a type I error of 0.05, power of 0.8, and a success rate (effect size) of 0.55, the required total sample size was 22 people. Considering a potential dropout of 20%, the sample size was inflated to at least 27 participants. Outliers were excluded by the Grubb’s test and data were classified as parametric or nonparametric by the Shapiro–Wilks test. For parametric variables, a paired Student T-Test was performed, and for nonparametric variables, Wilcoxon tests were applied. Linear and logistic regression tests were performed to verify all variables associated with the outcomes of interest. Analyses were performed using STATA^®^ 16-SE (Stata Corp. LCC, College Station, TX, USA) and GraphPad Prism 9.0 (GraphPad Software, La Jolla, CA, USA) software. Continuous parametric data were shown as mean ± standard deviation, and nonparametric as median and interquartile range. For all analyses, significance was determined as *p* < 0.05.

## 3. Results

### 3.1. Anthropometrics, Dietary Intake, Biochemistry, Cytokines, and Chemokines Modulation

The body mass index (BMI) and neck circumference reduced post-supplementation (Table 2). Body weight and other anthropometric parameters measured did not change after supplementation (Table S1). During the experimental period, there were no significant differences in dietary intake regarding energy (Kcal), carbohydrates, lipids, protein, and fiber consumption (Table S1). Concerning the plasmatic profile, there was an increase in total cholesterol and HDL-cholesterol post-supplementation. Additionally, post-supplementation there were higher levels of IgM, albumin, and creatinine, alongside an increase in TSH and thyroxine (Table 2). The other plasmatic parameters did not differ after supplementation, as shown in Table S2.

The plasma concentration for interleukin (IL)-8 reduced post-supplementation (Table 2). The levels of IL-1β (interleukin-1 beta), IL-6 (interleukin-6), IL-10 (interleukin-10), IL-12p70 (interleukin-12p70), TNF-α (tumor necrosis factor-alpha); CXCL10-IP10 (C-X-C motif chemokine ligand 10), CXCL9-MIG (C-X-C motif chemokine ligand 9-monokine induced by gamma interferon) and RANTES (regulated on activation normal T cell expressed and secreted), as well as the IL-6/IL-10 ratio and TNF-α/IL-10 ratio, did not differ (Figure S1).

### 3.2. Sleep, Mood, Physical Activity, and Quality of Life Effects

The Pittsburgh Sleep Quality Index (PSQI) global score as well as the (C1) Sleep Quality and (C7) Daytime Dysfunction components reduced post-supplementation (Table 2). The Brunel Mood Scale (BRUMS) dimension of Anger was reduced and the Confusion and Vigor dimensions were increased post-supplementation (Table 2). The Epworth Sleepiness Scale (ESS), the Mini-Sleep Questionnaire (MSQ-BR), physical activity level (IPAQ), and quality of life perception (WHOQoL-BREF) did not change over time (Table S3).

### 3.3. Gut Microbiota Reshaping

**Figure 2:**
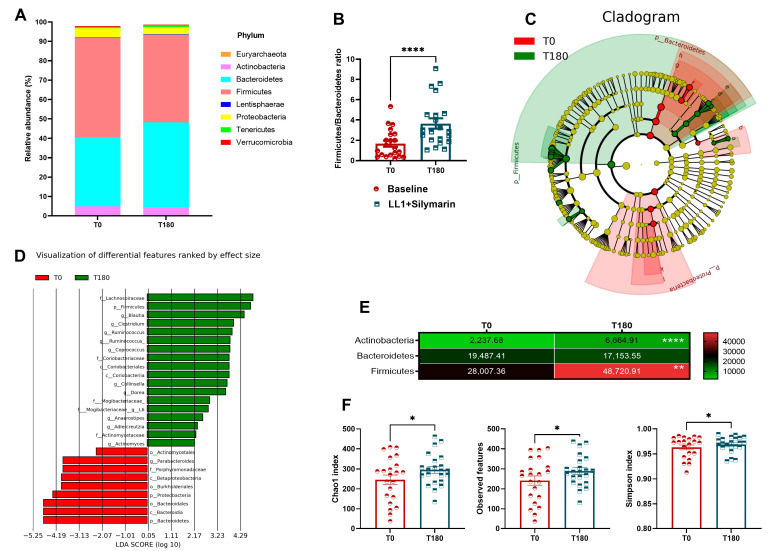
(**A**) Gut microbiota profile abundance in phyla. (**B**) Firmicutes/Bacteroidetes ratio. (**C**) Cladogram from LEfSe pre- and post-supplementation. (**D**) The logarithmic linear discriminant analysis (LDA) effect size (LEfSe) scores pre- and post-supplementation. (**E**) Heatmap of the modulated phyla. (**F**) Alpha (α) diversity indices of Chao1, Observed features, and Simpson index. Values are expressed as the percent of relative abundance (mean ± standard deviation). * *p* < 0.05. ** *p* < 0.01, **** *p* < 0.0001.

After supplementation, the gut microbiota exhibited increased alpha diversity, as reflected in the Chao1 index, Observed features, and Simpson index (Figure 2F). However, other alpha diversity metrics and all beta diversity metrics remained unchanged (Figure S2). The microbiota phyla composition was assessed pre- and post-supplementation (Figure 2A), revealing an increase in the Actinobacteria and Firmicutes post-supplementation (Figure 2E). Also, the Firmicutes/Bacteroidetes ratio was increased post-supplementation (Figure 2B). The T0 microbiota profile was enriched in Bacteroidetes and Proteobacteria phyla, while T180 was enriched mainly by Firmicutes and Bacteroidetes (Figure 2C). In the LEfSE analysis, potential alterations were more abundant and diverse in T180, reaching the genus level in the Linear Discriminant Analysis (LDA) score, highlighting the genus *Blautia* (Figure 2D).

**Figure 3:**
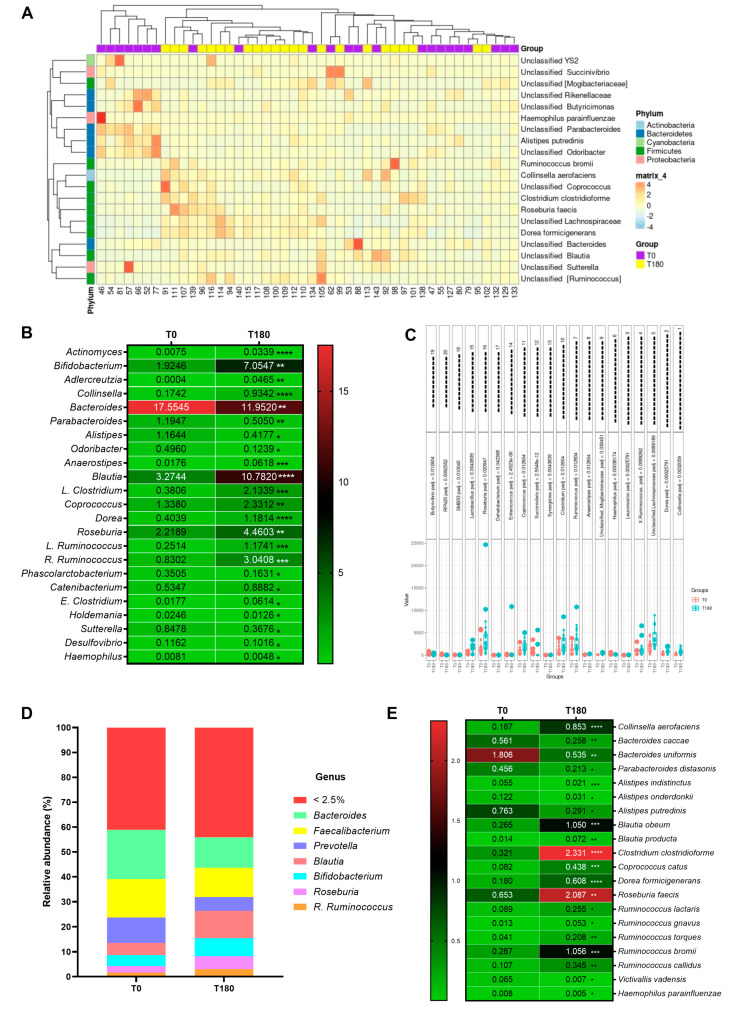
(**A**) Heatmap depicting taxonomic readings of volunteers’ microbiomes pre- and post-supplementation. (**B**) Heatmap of the microbial genera with statistical significance. (**C**) Differential expression analysis of the genera from gut microbiota. (**D**) Gut microbiota profile abundance in genera. (**E**) Heatmap of the microbial species with statistical significance. Values are expressed as the percentage of relative abundance (mean ± standard deviation). * *p* < 0.05, ** *p* < 0.01, *** *p* < 0.001, **** *p* < 0.0001.

The genera profile shows that supplementation improves diversity and the abundance of *Blautia* genera (Figure 3D). The heatmap helps to enhance the visualization of genus clusters with similar trends in expression, highlighting the increase in genera *Sutterella*, *Blautia*, and *Roseburia* in T180 (Figure 3A). In the genus, the post-supplementation reduced the abundance of *Bacteroides*, *Sutterella*, *Alistipes*, and *Odoribacter* while increasing the abundance of *Bifidobacterium*, *Collinsella*, *Blautia*, *Dorea*, *Roseburia*, *R. Ruminococcus* and *E. Clostridium* (Figure 3B). Differential expression analysis applied to the genus highlights the increase in the *Roseburia*, *Clostridium*, *Ruminococcus*, *Lachnospiraceae*, *Dorea*, and *Collinsella* genera post-supplementation (Figure 3C). The species abundance post-supplementation increased *Collinsella aerofaciens*, *Blautia obeum*, *Dorea formicigenerans*, *Clostridium clostridiforme*, *Roseburia faecis*, and *Ruminococcus bromii*, among others, alongside a reduction in *Bacteroide caccae*, *Alistipes indistinctus*, and other *Alistipes* species (Figure 3E).

### 3.4. Predictive Parameters of Microbiota Reshaping

The logistic regression analysis (Table 3) shows that body-weight loss is associated with enriched *Blautia producta*. The waist circumference in the middle abdomen (WC-mid) and WHtR reduction were associated with the improvement of the Bacteroidetes phylum. Hip circumference reduction was associated with the *Roseburia* genus increase. The WC-IC reduction was associated with an increase in Parabacteroides and the *Odoribacter* genus; WHR was associated with the Actinobacteria phylum and *Coprococcus* genus enrichment. The TNF-α/IL-10 ratio is associated with the genera *Anaerostipes* and *R. Ruminococcus*. The plasmatic IL-6 was associated with the *Prevotella*, *Oscillospira*, and *Bilophila* genera. The CXCL10-IP10 was associated with Firmicutes-related genera and species. The RANTES level and the BRUMS Anger dimension were associated with enriched α-diversity indices. The BRUMS Depression dimension was associated with the Bacteroidetes phylum and the *Lachnospira* genus, as well as the *Bacteroides uniformis* and *Alistipes onderdonkii* species. The BRUMS Vigor dimension was associated with *Ruminococcus bromii* and the BRUMS Fatigue dimension was associated with the *Streptococcus* genera.

The multiple linear regression shows (Table 4) that a reduction in the TNF-α/IL-10 ratio was associated with a decrease in *Bilophila*, *Bifidobacterium*, *Clostridium spiroforme*, and *Ruminococcus gnavus* and an increase in *Ruminococcus lactaris*. The IL-6 decrease was associated with a reduction in the *Catenibacterium* genus and an increase in *Ruminococcus Callidus*, *Faecalibacterium prausnitzii*, and *Parabacteroides distasonis*. The increase of IL-10 was associated with increased *Desulfovibrio* and decreased *Prevotella*. The decrease of IL-12p70 was associated with reduced species of *Catenibacterium* and elevated *Holdemania* genus, as well as *Faecalibacterium prausnitzii* and *Parabacteroides distasonis*. The reduced IL-8 was associated with increased α-diversity, Bacteroidetes phylum, *Ruminococcus bromii*, and *Ruminococcus gnavus* species, as well as the decrease in *Alistipes* genus abundance. RANTES reduction was associated with reduced Actinomyces and increased α-diversity (Faith’s PD). The reduction of the BRUMS Depression dimension was associated with *Desulfovibrio* and *Paraprevotella* reduction. The BRUMS Anger dimension decrease was positively associated with *Roseburia faecis* and negatively related to *Blautia producta.* The BRUMS Confusion dimension was associated with an increase in the *Alistipes* genus and a decrease in the Firmicutes phylum. The reduced score of PSQI (C2) Sleep Latency was associated with an increase in *Ruminococcus lactaris*. The PSQI (C3) Sleep Duration improvement was associated with *Faecalibacterium* increased abundance. The enhancement of PSQI (C4) Sleep Efficiency was associated with a reduction in the abundance of *Faecalibacterium prausnitzii* and *Alistipes indistinctus*. The improvement in the PSQI (C5) Sleep Disturbance score was associated with an increase in *Phascolarctobacterium*, *Faecalibacterium*, and *Odoribacter*, and the reduction of *Clostridium spiroforme* and *Bacteroides caccae*. The PSQI Global score improvement was related to the abundance of *Eubacterium biforme*, *Ruminococcus callidus*, *Alistipes putredinis*, and the Firmicutes/Bacteroidetes ratio. Other minor associations were verified and presented in Table S4.

## 4. Discussion

Integrative medicine strategies have grown in the wellness field due to the constant battle against mood disorders which directly impact overall health and quality of life [44]. This study brings to light the long-term supplementation of a nutraceutical blend, namely LL1 capsules—comprising essential minerals (selenium, magnesium, zinc, and chromium) and prebiotics (GOS, FOS, and beta-glucan)—associated with the herbal medicine Silymarin (*Silybum marianum* (L.) Gaertn) regarding its effect on gut microbiota composition, and its impact on inflammation, mood perception, and sleep quality. The formulation tested in this study was selected based on previous preclinical research that evaluated the effects of each single component on metabolic, endocrine, and microbiota modulation, compared with the LL1 individually and in combination with silymarin [45,46,47]. These studies demonstrated that the LL1 blend, particularly when combined with silymarin, had superior effects on these parameters compared to the individual components. Our research suggests that this enhanced efficacy may be due to nutrient synergy, which amplifies the beneficial effects on metabolism, endocrine function, and microbiota. Consequently, we proceeded to test this formulation in a human population, as detailed in this pilot clinical study.

The social isolation during the COVID-19 pandemic highlighted several mental health issues that had already been faced over the last few decades, uncovering this topic often neglected by society [48]. Undoubtedly, mental illness and mood disorders are serious illnesses that must receive medical monitoring, often associated with the use of medication. However, it is known that mood fluctuations are part of many people’s daily lives and can disrupt the routine even without a mental illness diagnosis. In this sense, mood changes can be influenced by several psychosocial, environmental, and physiological factors [49]. An important factor to be considered is the gut microbiota, because it regulates the production of essential factors for homeostasis, from inflammatory factors to neurotransmitters [50]. Therefore, the development of nutraceutical formulas that seek to balance the intestinal environment represents a promising tool for balancing mood and promoting well-being.

Notably, the supplementation promoted BMI and neck circumference reduction without changes in diet intake or exercise intervention. It is important to note that the statistical significance of these results was borderline, suggesting that further validation with a longer supplementation period may be needed to obtain more definitive outcomes. Nevertheless, the present result might be attributed to the synergistic interaction of the supplement composition enhancing metabolism [51]. Furthermore, considering the reduction in BMI without a corresponding decrease in body weight, along with the observed increase in creatinine levels, it is possible that the LL1 supplement contributed to lean mass gain. This aligns with preclinical results from an obese mice model, where LL1 supplementation led to increased lean body mass and reduced fat mass after just four weeks [45]. Pre-clinical tests of the nutraceutical formulation also showed an activation of mitochondrial biogenesis that can directly contribute to increased energy expenditure [45,46]. Furthermore, the LL1 prebiotics promote greater satiety and improved intestinal transit time [52], which may contribute to BMI reduction. The lack of exercise observed in our study sample highlights the supplement’s remarkable effect on BMI. Nevertheless, sedentarism might represent a trigger factor for mood disorders and sleep disturbance, impacting the quality of life [53].

The high-quality prebiotics offered might also be responsible for the improvement in the serum lipid profile driven by the increase in HDL-cholesterol levels [54] along with increased total cholesterol levels after supplementation. While an increase in total cholesterol might be considered a negative outcome due to its close and well-established relationship with a higher cardiovascular risk onset [55], our results indicate the only cholesterol fraction increased after supplementation was HDL. There were no changes in low-density lipoprotein (LDL), very low-density lipoprotein (VLDL), or non-HDL cholesterol levels, as shown in the supplementary material (Table S2). This suggests that the primary change in the cholesterol profile might be attributed to the increase in HDL, which is generally considered a positive effect. It is well known that prebiotics positively modulate the cholesterol profile, which might be due to gut microbiota production of short-chain fatty acids (SCFA) such as butyrate, which has been associated with reducing total cholesterol and LDL-cholesterol levels [56]. Our results indicate that the supplementation positively influenced endocrine modulation, affecting levels of TSH and thyroxine. Importantly, even though these parameters increased, they remained within the adequate clinical ranges, ruling out the possibility of hyperthyroidism or hypothyroidism. The literature highlights a strong connection between thyroid health and gut microbiota, since dysbiosis may significantly contribute to autoimmune and inflammatory thyroid conditions [57]. Additionally, the microbiota can affect the availability of essential micronutrients for the thyroid, such as iodine, iron, and copper, which are crucial for thyroid hormone synthesis, as well as selenium and zinc, which are also important for converting thyroxine (T4) to triiodothyronine (T3) [58]. Thus, our findings suggest that the microbiota reshape associated with the supplement composition, particularly with zinc and selenium, may enhance thyroid function in multiple ways. Also, endogenous subclinical hyperthyroidism may improve mood and quality of life perception [59], which can be related to our findings.

Furthermore, increased immune cell pro-inflammatory reactivity might be a trigger for mood disorders [60]. Populations under mood disorders present increased Toll-like receptors (TLRs) in peripheral monocytes and lymphocytes, higher activation of intracellular innate sensor NLRP3 (nucleotide-binding domain, leucine-rich–containing family, pyrin domain–containing-3) inflammasome and caspase-1 in blood cells, which correlate with increased serum levels of pro-inflammatory cytokines such as IL-1β, IL-8, TNF-α, IL-6 [4]. Our results have shown a significant reduction of IL-8 a pro-inflammatory chemokine crucial for angiogenesis and neutrophil attraction, and strongly related to tumorigenesis [61]. Recent research suggests that IL-8, among other cytokines, is involved in the gut–brain axis as well as the gut microbiota profile [62]. These results show that the microbiota plays a role in restoring inflammatory homeostasis and highlight the supplementation’s positive effects on mood and quality of life, in terms of endocrine and metabolic function, in agreement with previously published data [63].

Different forms of sleep disturbance commonly accompany mood disorders outlined in the Diagnostic and Statistical Manual of Mental Disorders (DSM-V) as symptoms of major depressive disorder and generalized anxiety disorder [64]. Our results have shown improvement in overall sleep quality and mood perception post-supplementation, which suggests modulation of the microbiota–gut–brain axis [65]. We hypothesize that the supplement components, such as zinc and magnesium, play an essential role as cofactors in serotonin synthesis from tryptophan amino acid [12,13]. Serotonin is a neurotransmitter related to the control of mood, sleep, and anxiety at a central level and with the modulation of gastrointestinal motility, glucose homeostasis, and adiposity in peripherical organs [66]. Serotonin is not only produced by the epiphysis (pineal gland) but also by the large intestine. Most peripheral serotonin comes from specialized enteroendocrine cells known as enterochromaffin cells, found throughout the gastrointestinal tract, sensing ingested nutrients and responding to gut microbiota and its metabolites. The interaction between gut microbiota and enterochromaffin cells is dynamic and has significant effects on host physiology and health [65]. Thus, the prebiotics supplemented by the LL1 capsules might help to keep a balanced gut microbiota favoring serotonin production.

Our results indicate that the supplementation was able to promote gut microbiota modulation from phyla to species levels, reshaping microbiota composition with an increase in microbiome diversity. Interestingly, the main microbiota phyla proportion changed with an increase in the Firmicutes phylum abundance. It was once thought that the Firmicutes phylum negatively impacted intestinal health due to its association with pathogenic bacteria and the development of chronic non-communicable diseases [67]. However, it is currently known that bacteria from the Firmicutes phylum are major producers of SCFA—propionate, butyrate, and acetoacetate—by the prebiotic’s fermentation in the intestinal lumen. The SCFA production plays a crucial role in intestinal barrier integrity maintenance. Additionally, SCFAs positively impact sleep and mood, since they can easily reach the central nervous system through the bloodstream [62]. The SCFAs, particularly butyrate, have neuroprotective properties, with Firmicutes being the main source of butyrate production in the gut microbiota, contributing significantly to overall health benefits [68]. Butyrate stimulates cell proliferation and differentiation in the dentate gyrus, boosting the expression of brain-derived neurotrophic factor (BDNF) and glial-derived neurotrophic factor (GDNF). Butyrate also provides an anti-inflammatory effect on the brain by inhibiting the production of TNF-α [69]. Therefore, the increased abundance of Firmicutes has a positive effect on the parameters analyzed in this study and can be justified by the increase in prebiotics such as FOS and GOS from LL1 capsules, which are high-quality substrates for microbiota fermentation, favoring the growth of these specific butyrate-producer colonies like the *Roseburia*, *Anaerostipes*, and *Coprococcus* genera.

It is interesting to note the associations of improvement in mood aspects like mental Confusion and Anger with enhanced Firmicute phyla and SCFAs-producer bacteria such as the *Roseburia* and *Blautia* genera. Similarly, the improvement in the PSQI global score and components of sleep duration, sleep efficiency, and sleep disturbance has been associated with butyrate and propionate producers’ genera like *Eubacterium*, *Coprococcus*, *Phascolarctobarterium*, and *Faecalibacterium*. In the literature, the increase in SCFAs-producer genera has been related to improvement in severe mood disorders (i.e., bipolar disorder, major depressive disorder, and schizophrenia), highlighting the crucial role of the gut microbiota in mood disorders and mental health [70]. Also, our results made clear that the supplementation improved the Bifidobacterium genera abundance, which is recognized as a BDNF stimulant and has been applied as probiotics for BDNF enhancement in mental illnesses like anxiety and depressive disorder [71]. Additionally, here we show an association of *Bifidobacterium* with improvement in the TNFα/IL-10 ratio and *Faecalibacterium* genus with IL6 and IL-12p70, displaying an anti-inflammatory potential which might reach the brain, as previously described in the literature [72].

Also, our results have presented improvement in the Actinobacteria phyla as well as the *R. Ruminococccus* and *L. Ruminococccus* genera post-supplementation, along with improvement in mood perception. A Mendelian randomization study analysis described a protective effect of gut microbiota *Actinobacteria*, *Bifidobacterium*, and *Ruminococcus* on major depressive disorder outcomes [73]. Our results show that *Ruminococcus lactaris* abundance was associated with improvement in sleep latency (C2) in the PSQI score, exerting a positive effect on sleep quality post-supplementation. Moreover, *Ruminococcus bromii* and *Ruminococcus gnavus* were associated with reduced pro-inflammatory cytokine IL-8, and an improved TNFα/IL-10 ratio was associated with *Ruminococcus gnavus* and *Ruminococcus lactaris* abundance, suggesting an anti-inflammatory relationship. Additionally, the alpha diversity indices (Chao1 and Observed features) were increased post-supplementation and associated with a reduction in pro-inflammatory cytokines (RANTES and IL-8) and improvement in the BRUMS Anger score, demonstrating the anti-inflammatory and mood-disorders-preventive effect of the supplementation. The improvement of alpha diversity in the gut microbiota has been recognized as a promising predictor for improvement in cognition and some neurological disease outcomes [74].

Furthermore, the role of flavonoids as prebiotic or “flavonobiotic” agents has recently been explored. Flavonoids are known for their beneficial effects on mental health due to their anti-inflammatory and antioxidant properties [75]. However, most flavonoids have low bioavailability as aglycon forms [76]. Consequently, most ingested flavonoids do not reach the bloodstream but are metabolized locally in the intestine, interacting with enterocytes and the intestinal microbiota [38,47]. These bioactive compounds have antibacterial effects, inhibiting pathogenic bacteria and promoting intestinal health and microbiota balance [77]. The silymarin extract may contribute positively to the modulation of intestinal microbiota post-supplementation. The prebiotic effect of silymarin might be attributed to its rich composition of flavonolignans such as silybin, isosilybin, silydianin, and silycristin [78]. These components, known as flavonobiotics, are metabolized by the microbiota and might influence the microbiota, besides the hepatoprotective effect known for the silymarin [79].

## 5. Conclusions

Thus, the presented results suggest that the supplementation of LL1 capsules and silymarin might be a promising tool for promoting microbiota modulation and improving the gut–brain axis. Also, the supplementation seems to specifically impact the Firmicutes phyla, promoting SCFA production and leading to the anti-inflammatory effect that might reach the central nervous system, helping to improve mood and sleep perception. Additionally, the microbiota reshape can lead to improved peripheric serotonin secretion, impacting well-being. Also, the association of mood and sleep quality variables with the gut microbiota reshape highlights its close relationship through the microbiota–gut–brain axis. It also highlights that the supplementation influenced the axis, improving the overall health perception, which is a promising effect for the nutraceutical formulation.

Moreover, it is important to consider that this clinical trial acknowledges inherent limitations in its experimental model, including small sample size, absence of a control or placebo group, SCFA analysis, and a lack of intermediary evaluating time-point. Regarding the small sample size, the COVID-19 pandemic significantly hindered our recruitment efforts, resulting in a high dropout rate and difficulties in enrolling new participants. These factors, beyond our control, unfortunately, impacted the research progress. While the absence of a placebo group in this clinical trial might seem unconventional, the literature indicates that common placebos in gut microbiota research, such as starch, resistant starch, cellulose, sugar, and maltodextrin, can interact with gut microbiota [21,22,23,24,25,26,27,28,29]. This interaction may bias results by altering the microbiota composition, producing metabolites utilized by intestinal cells, and entering the bloodstream. Given the lack of an ideal placebo that does not affect gut bacteria, this limitation might be considered minor. Additionally, the limited sample size is justified by a pilot study model and supported by the sample size calculation to ensure data reliability. Nevertheless, the findings presented here underwent meticulous scientific scrutiny to ensure their reliability and might constitute an initial effort to investigate the microbiota modulation related to better mood and sleep quality.

## Figures and Tables

**Table 1:** Supplements’ formulation components developed and tested in the present study.

LL1 (Long-Life 1) Capsules *	
Chromium (Cr)	75 mcg
Zinc (Zn)	26 mg
Magnesium (Mg)	63.4 mg
Fructooligosaccharide (FOS)	45%
Selenium	140 mcg
Galactooligosaccharide (GOS)	10%
1.3/1.6-(β-glycosidic bonds) yeast β-glucans (*Saccharomyces cerevisiae*)	250 mg
**Silymarin Capsule**	
Silymarin extract (*Silybum marianum* (L.) Gaertn.) ^#^	150 mg

* four capsules of dosage; ^#^ *Silybum marianum* seed extract standardized with 38.6% of silymarin.

**Table 2:** Demographic, anthropometric, plasmatic profile, sleep quality, and mood before and after supplementation.

LL1 + Silymarin					
Sample size (M/F)	22 (7/15)				
Age (years)	58.54 ± 5.65				
Height (m)	1.62 ± 0.10				
Anthropometrics					
	T0		T180		
	Mean ± SD		Mean ± SD		*p*
BMI (kg/m^2^)	28.9 ± 0.67		28.3 ± 0.76		0.0465
Neck (cm)	36.3 ± 0.70		35.7 ± 0.73		0.0049
Plasmatic profile					
Total Cholesterol (mg/dL)	194 ± 7.81		208 ± 7.19		0.0255
HDL-C (mg/dL)	45.7 ± 2.3		51.8 ± 2.61		0.0002
IgM (mg/dL)	104 ± 10.4		107 ± 10.4		0.0106
Albumin (g/dL)	4.65 ± 0.043		4.93 ± 0.052		0.0001
Creatinine (mg/dL)	0.83 ± 0.36		0.90 ± 0.04		0.0007
TSH (mUI/L)	2.02 ± 0.243		2.72 ± 0.352		0.0479
Thyroxine (ng/dL)	0.981 ± 0.045		1.36 ± 0.043		0.0001
IL-8 (pg/mL)	0.859 ± 0.231		0.422 ± 0.156		0.0161
	T0		T180		
	Mean ± SD	CI 95%	Mean ± SD	CI 95%	*p*
Pittsburgh Sleep Quality Index (PSQI)					
PSQI Global score	6.39 ± 3.12	5.04–7.74	5.17 ± 2.77	3.97–6.37	0.0198
(C1) Sleep Quality	1.17 ± 0.65	0.89–1.46	0.83 ± 0.57	0.57–1.08	0.0078
(C2) Sleep Latency	1.09 ± 0.85	0.72–1.45	0.78 ± 0.16	0.44–1.13	
(C3) Sleep Duration	1.17 ± 0.58	0.93–1.42	1.30 ± 0.87	0.93–1.68	-
(C4) Sleep Efficiency	0.43 ± 0.73	0.12–0.75	0.43 ± 0.89	0.05–0.82	-
(C5) Sleep Disturbance	1.13 ± 0.55	0.89–1.37	0.96 ± 0.47	0.75–1.16	-
(C6) Sleep Medication	0.26 ± 0.75	−0.06–0.59	0.13 ± 0.62	−0.14–0.40	-
(C7) Daytime Dysfunction	1.35 ± 1.43	0.73–1.97	0.68 ± 0.65	0.39–0.97	0.0042
Brunel Mood Scale (BRUMS)					
BRUMS total score	20.14 ± 1.31	17.40–22.89	21.62 ± 1.46	18.56–24.68	-
Tension	3.90 ± 1.09	3.41–4.40	4.41 ± 1.71	3.65–5.17	-
Depression	2.38 ± 1.66	1.63–3.14	3.77 ± 3.24	2.34–5.21	-
Anger	3.19 ± 2.27	2.16–4.22	1.90 ± 1.61	1.17–2.64	0.0082
Confusion	3.90 ± 1.04	3.43–4.38	4.62 ± 1.47	3.95–5.29	0.0199
Vigor	3.48 ± 1.66	2.72–4.23	4.29 ± 1.55	3.58–4.99	0.0420
Fatigue	3.10 ± 1.34	2.49–3.70	3.24 ± 1.30	2.65–3.83	-

BMI: body mass index; HDL-C: high-density lipoproteins cholesterol; TSH: thyroid-stimulating hormone.

**Table 3:** Logistic regression analysis from gut microbiota association with clinical-demographic characteristics after supplementation.

	%	R^2^	IC 95% Min–Max		*p*
Body weight (kg)	*Blautia producta*	0.71	0.22	1.21	0.009
WC-mid (cm)	Bacteroidetes	26.66	2.3	308.01	0.009
Hip (cm)	*Roseburia*	23.33	1.99	273.29	0.012
WC-IC (cm)	*Parabacteroides*	17.5	1.59	191.89	0.019
	*Odoribacter*	19.99	1.67	238.62	0.018
WHR	Actinobacteria	0.15	0.011	2.055	0.155
	*Coprococcus*	12.01	1.58	91.08	0.016
WHtR	Bacteroidetes	17.5	1.59	191.89	0.019
TNF-α/IL-10 ratio	*Anaerostipes*	56.01	2.92	1071.63	0.008
	*R. Ruminococcus*	15.75	1.42	174.24	0.025
IL-6 (pg/mL)	*Prevotella*	10.5	1.11	98.91	0.04
	*Oscillospira*	7.11	1.08	46.44	0.04
	*Bilophila*	13.5	1.19	152.21	0.035
RANTES (pg/mL)	α-diversity chao1 index	12.01	1.11	128.83	0.04
	α-diversity Faith’s PD	12.01	1.11	128.83	0.04
	α-diversity Obs features	12.01	1.11	128.83	0.04
CXCL10/IP-10 (pg/mL)	*Oscillospira*	7.11	1.08	46.44	0.04
	*R. Ruminococcus*	7.11	1.08	46.44	0.04
BRUMS Anger	α-diversity chao1 index	21.01	1.5	293.25	0.024
	α-diversity Obs features	21.01	1.5	293.25	0.024
	α-diversity Shanon entropy	21.01	1.5	293.25	0.024
BRUMS Depression	Bacteroidetes	32.01	2.39	427.74	0.009
	*Bacteroides uniformis*	8.166	1.027	64.93	0.047
	*Alistipes onderdonkii*	30.01	1.471	611.79	0.027
	*Lachnospira*	0.122	0.015	0.973	0.047
BRUMS Vigor	*Ruminococcus bromii*	24.01	1.14	505.19	0.041
BRUMS Fatigue	*Streptococcus*	14.01	1.13	172.64	0.039

WC-mid: waist circumference in the middle abdomen; WHR: waist-to-hip ratio; WHtR: waist-to-height ratio.

**Table 4:** Multiple linear regression analysis from gut microbiota association with clinical biomarkers after supplementation.

Variable	(%)	Coef	IC 95% Min–Max		*p*
↓TNF-α/IL-10 ratio	*Bilophila*	−4.4251	−7.7851	−1.0650	0.016
	*Bifidobacterium*	−0.1032	−0.1740	−0.0323	0.012
	*Clostridium spiroforme*	−3.2441	−4.9353	−1.5529	0.009
	*Ruminococcus gnavus*	−0.3283	−0.5890	−0.0677	0.022
	*Ruminococcus lactaris*	2.6987	0.1400	5.2573	0.042
↓IL-6 (pg/mL)	*Catenibacterium*	−0.1599	−0.2459	−0.0739	0.003
	*Ruminococcus callidus*	0.7058	0.2054	1.2062	0.012
	*Faecalibacterium prausnitzii*	0.4652	0.2876	0.6428	<0.0001
	*Parabacteroides distasonis*	0.1206	0.0121	0.2291	0.032
↑IL-10 (pg/mL)	*Desulfovibrio*	1.3290	0.0201	2.6379	0.047
	*Prevotella*	−0.9742	−1.6373	−0.3110	0.008
↓IL-12p70 (pg/mL)	*Holdemania*	0.8647	0.0005	1.7289	0.05
	*Catenibacterium*	−0.1341	−0.2282	−0.0401	0.01
	*Faecalibacterium prausnitzii*	0.4129	0.1619	0.6639	0.003
	*Parabacteroides distasonis*	0.1785	0.0859	0.2711	0.001
↓IL-8 (pg/mL)	*Ruminococcus bromii*	5.4948	2.0311	8.9585	0.012
	Bacteroidetes	1.6105	0.0040	3.2169	0.05
	*Alistipes*	−0.814	−1.590	−0.039	0.041
	α-diversity Faith’s PD	2.021	0.258	3.784	0.027
	*Ruminococcus gnavus*	0.280	0.028	0.532	0.033
↓RANTES (pg/mL)	*Actinomyces*	−0.802	−1.253	−0.351	0.003
	α-diversity Faith’s PD	−1.381	−2.752	−0.011	0.048
↓Depression (BRUMS)	*Desulfovibrio*	−1.693	−2.670	−0.715	0.005
	*Paraprevotella*	−1.172	−1.636	−0.709	0.002
↓Anger (BRUMS)	*Roseburia faecis*	0.302	0.085	0.519	0.010
	*Blautia producta*	−0.303	−0.597	−0.009	0.045
↓Confusion (BRUMS)	*Alistipes*	0.182	0.011	0.353	0.038
	Firmicutes	−0.220	−0.388	−0.053	0.013
↓PSQI (C2) Sleep Latency	*Ruminococcus lactaris*	0.142	0.116	0.167	0.002
↓PSQI (C3) Sleep Duration	*Faecalibacterium*	0.279	0.011	0.548	0.042
↓PSQI (C4) Sleep Efficiency	*Faecalibacterium prausnitzii*	−0.608	−0.785	−0.431	0.002
	*Alistipes indistinctus*	−0.127	−0.182	−0.072	0.022
↓PSQI (C5) Sleep Disturbance	*Phascolarctobacterium*	0.136	0.044	0.229	0.009
	*Faecalibacterium*	0.410	0.224	0.596	<0.0001
	*Clostridium spiroforme*	−0.378	−0.511	−0.246	0.001
	*Bacteroides caccae*	−0.102	−0.191	−0.013	0.030
↓PSQI Global score	*Eubacterium biforme*	0.507	0.128	0.886	0.014
	*Ruminococcus callidus*	2.352	0.876	3.829	0.006
	*Coprococcus catus*	1.094	0.182	2.007	0.022
	F/B ratio	0.785	0.102	1.468	0.027

## Data Availability

The data supporting this study’s findings are available upon request from the corresponding author. However, these data were used under license for the current investigation and are not publicly available due to restrictions. The datasets generated and/or analyzed during the current study are available in the GenBank^®^ repository, Bioproject PRJNA941000. Link: https://www.ncbi.nlm.nih.gov/sra/PRJNA941000 (release date: 30 June 2025).

## Supplementary Materials

The following supporting information can be downloaded at: https://www.mdpi.com/article/10.3390/nu16183049/s1, Table S1. Anthropometric data and diet intake before and after the supplementation. Table S2. Serum parameters analyses before and after the supplementation. Figure S1. Cytokines and chemokines in plasma after 180 days of supplementation. (A) IL-1β; (B) IL-6; (C) IL-10; (D) IL-12p70; (E) TNF-α; (F) IL-6/IL-10 ratio; (G) TNF-α/IL-10 ratio; (H) CXCL10/IP-10; (I) CXCL9/MIG; (J) RANTES. Table S3. Characteristics of volunteers’ sleep quality, daytime sleepiness, quality of life, and physical activity level. Figure S2. Gut microbiota diversity indices post-supplementation. (A) Alpha (α) diversity indices of Shannon entropy, Pielou’s evenness, and Faith’s Phylogenetic diversity (PD). (B) beta (β)-diversity of Bray-Curtis distance, Jaccard distance, and Unweighted and Weighted UniFrac distances. Values are expressed as median, min, and max. Table S4. Regression analysis from gut microbiota association with clinical-demographic characteristics after supplementation.
