# Supplementary material for: Nutraceutical Capsules LL1 and Silymarin Supplementation Act on Mood and Sleep Quality Perception by Microbiota–Gut–Brain Axis: A Pilot Clinical Study
Source: Nutrients. 2024 Sep 10;16(18):3049. doi: 10.3390/nu16183049 (PMC11435014; doi:10.3390/nu16183049)
Supplement: Supplementary file 1 [file nutrients-16-03049-s001.zip › nutrients-3161587-supplementary.pdf]

Table S1. Anthropometric data and diet intake before and after the supplementation.

	LL1 + silymarin		
	**T0**	**T180**	
	Mean ± SD	Mean ± SD	*p*
	**Anthropometrics**		
**Body mass (kg)**	76.1±2.79	75.4±2.81	
**WC-mid (cm)**	93.2±2.1	92±2.19	-
**Hip (cm)**	107±1.6	107±1.60	-
**WC-IC (cm)**	104±1.89	103±1.91	-
**WHR**	0.86±0.013	0.87±0.013	-
**WHtR**	0.575±0.011	0.569±0.012	-
	**Dietary Intake**		
**Energy (Kcal)**	1758±69	1839±107	-
**Carbohydrates (g)**	235±9.78	252±15.4	-
**Fiber (g)**	13.1±1.27	12.5±1.11	-
**Lipids (g)**	57.9±3.76	61.9±5.64	-
**Proteins (g)**	74.5±3.64	78±6.39	-

WC-mid: waist circumference in middle abdomen; WC-IC: waist circumference in iliac crest; WHR: waist-to-hip ratio, WHtR: waist-to-height ratio.

Table S2. Serum parameters analyses before and after the supplementation.

	LL1 + silymarin		
	**T0**	**T180**	
	Mean ± SD	Mean ± SD	*p*
**Glucose tolerance**			
**Glycemia (mg/dL)**	97.8±3.03	92.6±1.87	-
**Insulin (mU/L)**	12.6±1.27	14.6±1.69	-
**HbA1c (%)**	5.49±0.088	5.55±0.101	-
**HOMA-IR**	3.07±0.35	3.36±0.43	-
**Serum lipids profile**			
**LDL-C (mg/dL)**	123±7.2	132±6.53	-
**VLDL-C (mg/dL)**	25±1.43	24.2±1.49	-
**Non-HDL-C (mg/dL)**	148±7.59	157±7.04	-
**Triglycerides (mg/dL)**	130±9.03	130±9.94	-
**Immunoglobulins**			
**IgA (mg/dL)**	198±15.8	201±14.9	-
**IgG (mg/dL)**	1088±38.9	1091±39.8	-
**Serum protein profile**			
**Total protein (g/dL)**	7.25±0.071	7.3±0.078	-
**C-Reactive protein (mg/dL)**	0.151±0.031	0.182±0.029	-
**Liver and Kidney function markers**			
**AST (U/L)**	18.4±0.081	20±1.33	-
**ALT (U/L)**	13.2±1.07	15.3±1.3	-
**AST/ALT ratio**	1.48±0.09	1.31±0.07	-
**Alkaline phosphatase (U/L)**	76.8±3.69	79.1±3.9	-
***Gamma*-GT (U/L)**	19.4±1.61	20±1.47	-
**Serum hormones**			
**Cortisol (ug/dL)**	10.8±0.78	11.1±0.81	-
**Cortisol/ C-RP ratio**	122.7±22.12	105.4±19.55	-

AST: aspartate aminotransferase; ALT: alanine aminotransferase.

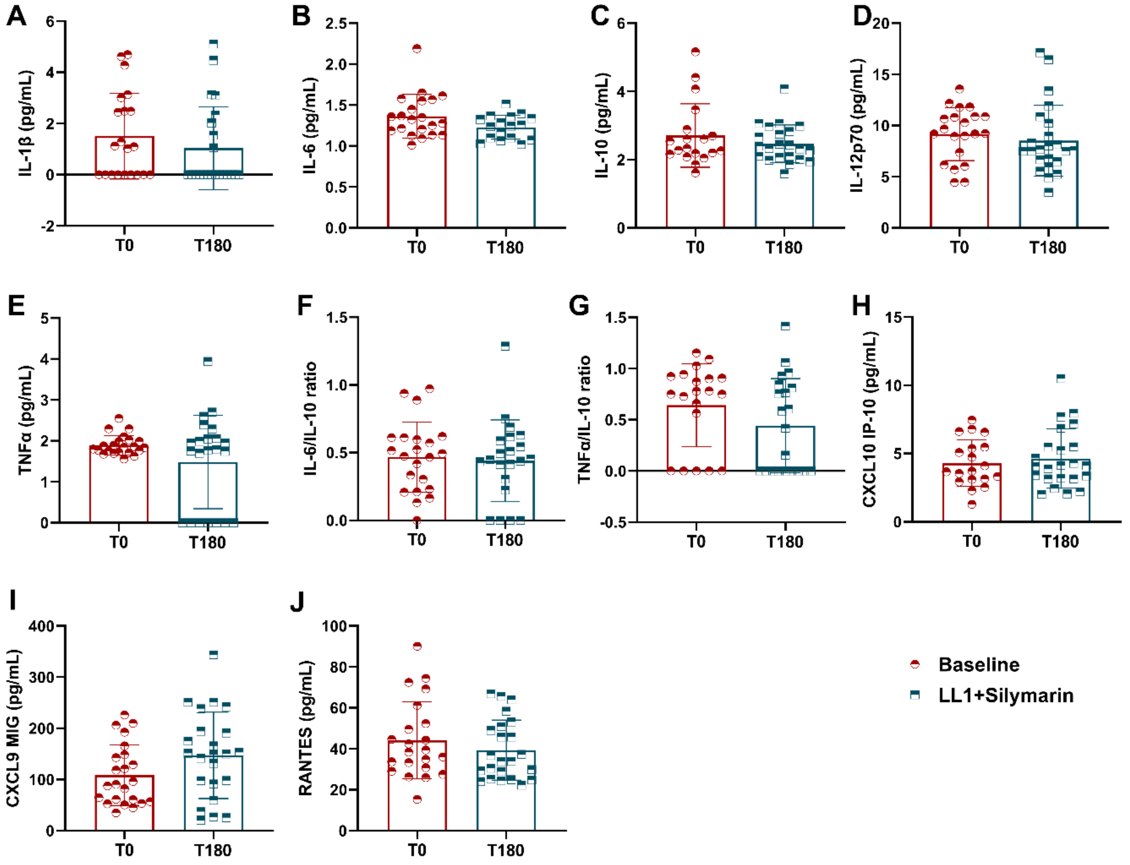

Figure S1. Cytokines and chemokines in plasma after 180 days of supplementation. **(A)** IL-1β; **(B)** IL-6; **(C)** IL-10; **(D)** IL-12p70; **(E)** TNF-α; **(F)** IL-6/IL-10 ratio; **(G)** TNF-α/IL-10 ratio; **(H)** CXCL10/IP-10; **(I)** CXCL9/MIG; **(J)** RANTES.

Table S3. Characteristics of volunteers sleep quality, daytime sleepiness, quality of life, and physical activity level.

	LL1 + silymarin				
	T0		T180		
	Mean ± SD	CI 95%	Mean ± SD	CI 95%	*p*
**Epworth Sleepiness Scale (ESS)**					
**ESS Total Score**	11.6±1.34	8.79- 14.3	11.5±1.05	9.38 - 13.7	-
**Mini-Sleep Questionnaire (MSQ-BR)**					
**MSQ-BR Score**	28.1±1.76	24.5 - 31.9	29.9±1.74	26.3 - 33.7	-
**WHO QoL-BREF**					
**Overall QoL and General health**	3.80 ± 0.54	3.57 – 4.04	3.65 ± 0.75	3.33 – 3.97	-
**Physical domain**	3.69 ± 0.55	3.45 – 3.92	3.61 ± 0.52	3.39 – 3.83	-
**Psychological domain**	3.69 ± 0.55	3.45 - 3.93	3.60 ± 0.56	3.36 - 3.84	-
**Social relationships**	3.70 ± 0.41	3.52 - 3.88	3.64 ± 0.63	3.36 - 3.92	-
**Environment**	3.46 ± 0.38	3.30 - 3.63	3.4 5± 0.46	3.25 - 3.65	-
**International Physical Activity Questionnaire (IPAQ)**					
	T0		T180		
	n (%)		n (%)		*p*
**Sedentary**	8 (36.5)		7 (32)		-
**irregularly active**	7 (32)		8 (36.5)		-
**Moderate active**	6 (27)		6 (27)		-
**Highly active**	1 (4.5)		1 (4.5)		-

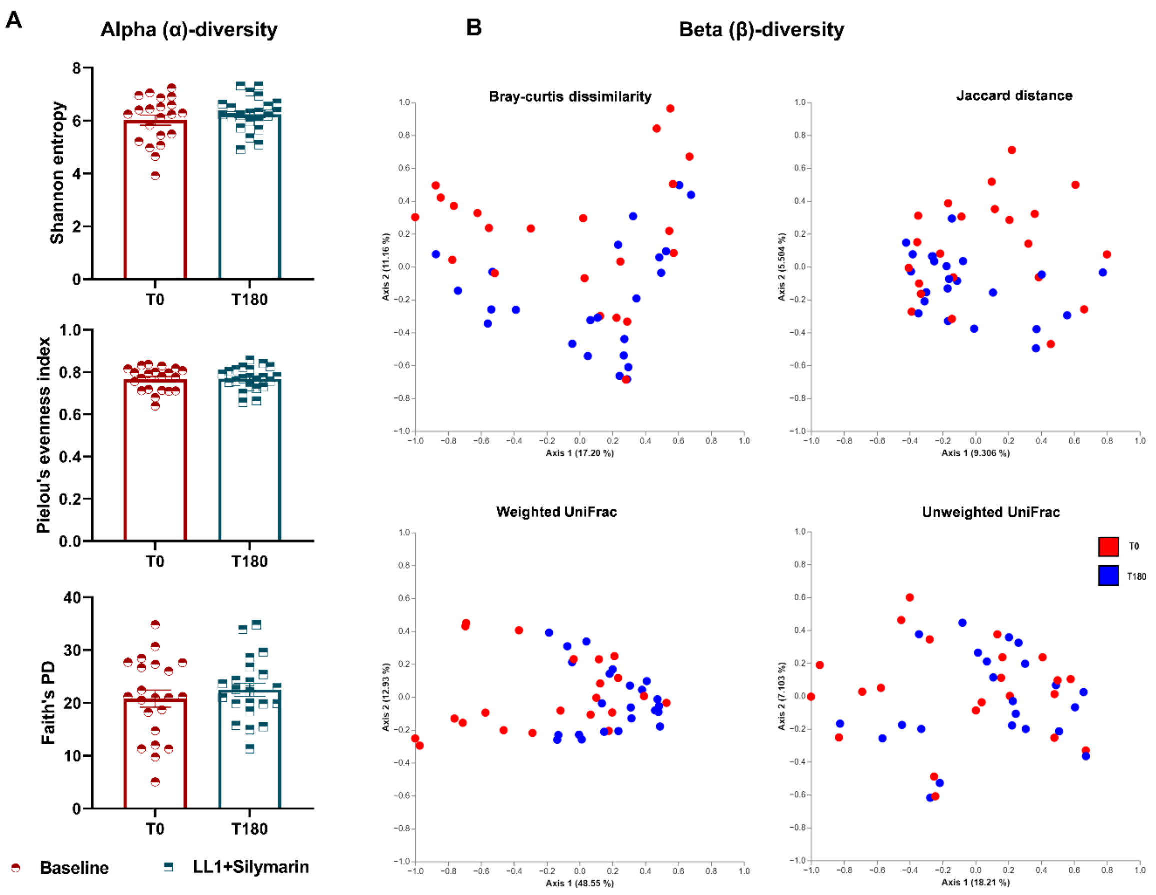

Figure S2. Gut microbiota diversity indices post-supplementation. **(A)** Alpha (α) diversity indices of Shannon entropy, Pielou's evenness and Faith's Phylogenetic diversity (PD). **(B)** beta (β)-diversity of Bray-Curtis distance, Jaccard distance, Unweighted and Weighted UniFrac distances. Values are expressed as median, Min, and Max.

Table S4. Regression analysis from gut microbiota association with clinical-demographic characteristics after supplementation.

		Logistic regression			
	%	R^2	IC 95% min	IC95% max	*p*
**WC-mid (cm)**	*Clostridium clostridioforme*	0.042	0.003	0.501	0.012
	Lachnospira	0.0375	0.003	0.43	0.009
	Coprococcus	0.0375	0.003	0.43	0.009
**WC-IC (cm)**	*Streptococcus*	0.0571	0.005	0.62	0.019
	Coprococcus	0.0571	0.005	0.62	0.019
**WHtR**	*Lachnospira*	0.05	0.005	0.626	0.019
	Clostridium clostridioforme	0.06	0.005	0.74	0.028
**TNF-α/IL-10 ratio**	*Parabacteroides*	0.06	0.005	0.7	0.025
**IL-12p70 (pg/mL)**	*Lachnobacterium*	0.057	0.005	0.626	0.019
	Roseburia	0.057	0.005	0.626	0.019
**TNF-α (pg/mL)**	*Lachnobacterium*	0.057	0.005	0.626	0.019
	Roseburia	0.057	0.005	0.626	0.019
**RANTES (pg/mL)**	*Collinsella*	0.066	0.0059	0.745	0.028
**BRUMS Depression**	*Ruminococcus lactaris*	0.041	0.0019	0.877	0.041
	Coprococcus	0.031	0.0023	0.417	0.009
	R. ruminococcus	0.071	0.0078	0.649	0.019
		**Multiple linear regression**			
**Body weight (kg)**	*Streptococcus*	0.00247	0.00086	0.00408	*0.005*
**BMI (kg/m²)**	*Streptococcus*	0.0025	0.0009	0.0041	*0.005*
**Neck (cm)**	α-diversity Observed features	-0.0358	-0.0691	-0.0025	*0.036*
	α-diversity Faith's PD	-0.0467	-0.0882	-0.0051	*0.03*
	α-diversity chao1 index	-0.0356	-0.0689	-0.0024	*0.037*
	Haemophilus parainfluenzae	-0.0002	-0.0003	-0.00001	*0.04*
	Victivallis vadensis	0.0058	0.0016	0.0101	*0.022*
**WC-mid (cm)**	*Phascolarctobacterium*	-0.0460	-0.0722	-0.0198	*0.002*
	Paraprevotella	-0.0291	-0.0542	-0.0041	*0.028*
**Hip (cm)**	*Phascolarctobacterium*	-0.0177	-0.0288	-0.0067	*0.004*
**WC-IC (cm)**	*Desulfovibrio*	-0.0614	-0.1047	-0.0180	*0.01*
	Holdemania	-0.0278	-0.0553	-0.0003	*0.048*
	Phascolarctobacterium	-0.0290	-0.0481	-0.0099	*0.006*
	Paraprevotella	-0.0379	-0.0676	-0.0083	*0.019*
**WHR**	*Slackia*	-0.0046	-0.0089	-0.0003	*0.039*
**WHtR**	*Phascolarctobacterium*	-0.0460	-0.0722	-0.0198	*0.002*
	Paraprevotella	-0.0291	-0.0542	-0.0041	*0.028*
**TNF-α/IL-10 ratio**	*Haemophilus parainfluenzae*	-0.0213	-0.0277	-0.0149	*0.005*

**IL-10 (pg/mL)**	*Slackia*	0.0423	0.0026	0.0819	*0.04*
	Haemophilus parainfluenzae	-0.0031	-0.0056	-0.0005	*0.026*
**IL-12p70 (pg/mL)**	*Ruminococcus*	-0.0810	-0.1472	-0.0148	*0.019*
**TNF-α (pg/mL)**	*Eubacterium biforme*	0.076	0.007	0.144	*0.036*
**CXCL8 (pg/mL)**	*Lactobacillus*	0.071	0.044	0.098	*0.002*
**RANTES (pg/mL)**	*Akkermansia*	-0.067	-0.117	-0.016	*0.019*
**CXCL9/MIG (pg/mL)**	*L. Clostridium*	-0.056	-0.070	-0.043	*<0.0001*
**Tension (BRUMS)**	*L. Clostridium*	-0.018	-0.023	-0.013	*<0.0001*
**Anger (BRUMS)**	*Akkermansia*	-0.038	-0.070	-0.007	*0.025*
	Anaerostipes	-0.062	-0.117	-0.008	*0.031*
	Streptococcus	-0.032	-0.055	-0.010	*0.009*
**Vigor (BRUMS)**	*L. Clostridium*	-0.009	-0.018	-0.001	*0.042*
**Confusion (BRUMS)**	*Catenibacterium*	-0.033	-0.056	-0.009	*0.011*
	Coprococcus	0.030	0.005	0.056	*0.021*
	Haemophilus parainfluenzae	0.003	0.001	0.005	*0.016*
	Ruminococcus gnavus	-0.047	-0.088	-0.005	*0.03*
	Parabacteroides distasonis	0.048	0.018	0.077	*0.004*
**MSQ-BR Score**	*Akkermansia*	0.027	0.005	0.048	*0.024*
	Alistipes onderdonkii	-0.036	-0.064	-0.007	*0.019*
	Alistipes indistinctus	-0.065	-0.109	-0.021	*0.008*
**ESS Total Score**	*Haemophilus parainfluenzae*	0.003	0.001	0.006	*0.026*
**PSQI (C3) Sleep Duration**	*Alistipes onderdonkii*	-0.047	-0.078	-0.016	*0.007*
**PSQI Global score**	*Akkermansia*	0.027	0.004	0.050	*0.028*
	Bifidobacterium	-0.080	-0.131	-0.029	*0.006*
